# Supplementary material for: Causal Relationship between Aging and Anorexia Nervosa: A White-Matter-Microstructure-Mediated Mendelian Randomization Analysis
Source: Biomedicines. 2024 Aug 16;12(8):1874. doi: 10.3390/biomedicines12081874 (PMC11351342; doi:10.3390/biomedicines12081874)
Supplement: Supplementary file 1 [file biomedicines-12-01874-s001.zip › Supplementary Figure/Figure S4/Singe SNP plot of MR analysis for TL to ubm-b-2032.pdf]

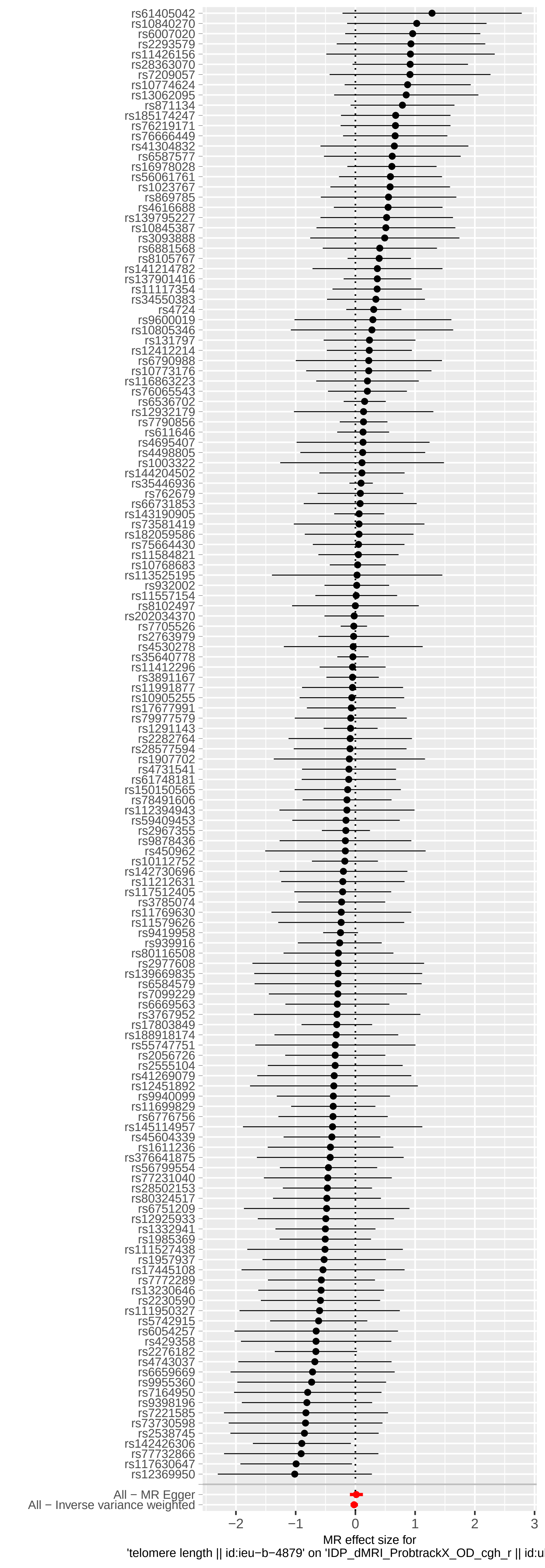
rs61405042
rs10840270
rs6007020
rs2293579
rs11426156
rs28363070
rs7209057
rs10774624
rs13062095
rs871134
rs185174247
rs76219171
rs76666449
rs41304832
rs6587577
rs16978028
rs56061761
rs1023767
rs869785
rs4616688
rs139795227
rs10845387
rs3093888
rs6881568
rs8105767
rs141214782
rs137901416
rs11117354
rs34550383
rs4724
rs9600019
rs10805346
rs131797
rs12412214
rs6790988
rs10773176
rs116863223
rs76065543
rs6536702
rs12932179
rs7790856
rs611646
rs4695407
rs4498805
rs1003322
rs144204502
rs35446936
rs762679
rs66731853
rs143190905
rs73581419
rs182059586
rs75664430
rs11584821
rs10768683
rs113525195
rs932002
rs11557154
rs8102497
rs202034370
rs7705526
rs2763979
rs4530278
rs35640778
rs11412296
rs3891167
rs11991877
rs10905255
rs17677991
rs79977579
rs1291143
rs2282764
rs28577594
rs1907702
rs4731541
rs61748181
rs150150565
rs78491606
rs112394943
rs59409453
rs2967355
rs9878436
rs450962
rs10112752
rs142730696
rs11212631
rs117512405
rs3785074
rs11769630
rs11579626
rs9419958
rs939916
rs80116508
rs2977608
rs139669835
rs6584579
rs7099229
rs6669563
rs3767952
rs17803849
rs188918174
rs55747751
rs2056726
rs2555104
rs41269079
rs12451892
rs9940099
rs11699829
rs6776756
rs145114957
rs45604339
rs1611236
rs376641875
rs56799554
rs77231040
rs28502153
rs80324517
rs6751209
rs12925933
rs1332941
rs1985369
rs111527438
rs1957937
rs17445108
rs7772289
rs13230646
rs2230590
rs111950327
rs5742915
rs6054257
rs429358
rs2276182
rs4743037
rs6659669
rs9955360
rs7164950
rs9398196
rs7221585
rs73730598
rs2538745
rs142426306
rs77732866
rs117630647
rs12369950
All − MR Egger
All − Inverse variance weighted
−2
−1
0
1
2
3
MR effect size for
'telomere length || id:ieu−b−4879' on 'IDP_dMRI_ProbtrackX_OD_cgh_r || id:u
